# Nerve Grafts in Breast Reconstruction: A Narrative Review

**DOI:** 10.1155/tbj/1035158

**Published:** 2025-12-31

**Authors:** Yan Shen, Juan Zhang, Yihua Liu, Haifeng Cai

**Affiliations:** ^1^ Department of Breast Surgery, Tangshan People’s Hospital, Tangshan, 063000, Hebei, China; ^2^ Department of General Medical Subjects, Ezhou Central Hospital, Ezhou, 436000, Hubei, China

**Keywords:** breast cancer, breast reconstruction, nerve grafting

## Abstract

Breast reconstruction post‐mastectomy restores breast aesthetics and significantly enhances patients’ self‐confidence and psychological well‐being. However, despite the effectiveness of current breast reconstruction techniques in restoring the aesthetic appearance of the breast, many patients continue to experience the loss of breast sensation following surgery, particularly the loss of nipple sensitivity, which significantly impacts postoperative quality of life. To address this issue, the use of nerve grafting techniques in breast reconstruction is increasingly being recognized and explored. This review summarizes the research progress of nerve grafting in breast reconstruction, highlighting its clinical applications, technical challenges, and future directions.

## 1. Introduction

Breast cancer is a pervasive malignancy among women that requires surgical management as the primary treatment option. Breast reconstruction plays a crucial role in the postoperative management of breast cancer patients, aiming to restore both the appearance and psychological well‐being of individuals. Nipple‐sparing mastectomy (NSM) with implant‐based reconstruction has become increasingly favored by surgeons and patients due to its favorable aesthetic outcomes, low complication rates, and reduced psychological impact of mastectomy [1]. Traditional reconstruction techniques often result in postoperative sensory loss, significantly affecting the quality of life of the patients. In a prospective longitudinal study of 259 postoperative breast cancer patients conducted by K. M. Flowers, approximately half of the participants reported experiencing numbness following surgery [2]. A prospective longitudinal study that assessed body image, quality of life, and pain in breast cancer surgery patients before and at 30, 60, and 90 days postoperatively revealed significant differences in body image between the preoperative and 30‐day postoperative assessments, along with changes in sexual function and satisfaction [3]. Recent advances in nerve repair have facilitated the integration of nerve grafting into breast reconstruction, marking a significant advancement in restoring sensory function to reconstructed breasts [4].

## 2. Neuroanatomy of the Breast

There has been extensive debate regarding the distribution of nerves innervating the breast, particularly those associated with the nipple–areola complex (NAC). The nerve distribution is primarily known through autopsy studies. The sensory innervation of the breast stems from the lateral cutaneous branches (LCBs) and anterior cutaneous branches (ACBs) of the 2nd to 6th intercostal nerves (ICNs) and, to a lesser extent, the supraclavicular nerve [5–7]. Schlenz et al. dissected 28 female cadavers and found that the NACs were always innervated by the LCBs and ACBs of the 3rd, 4th, and 5th ICNs. The most constant innervation pattern was by the 4th LCBs (79%) and by the 3rd and 4th ACBs (57%) [8]. Subsequent studies revealed that the nipples are innervated by a broad range of nerves, almost always encompassing both the ACB and the LCB of the 3rd to 5th ICNs [5, 7–9]. All studies refer to the LCB‐innervated nipple of the 4th ICN. Recent studies have demonstrated that ACB of the 4th ICN innervates the largest area of skin in the healthy breast, followed by the ACB of the 3rd ICN as the second largest area. The 4th ICN consistently supplies the NAC and represents the optimal reinnervation target [10].

However, these controversial reports may be attributed to difficulties in dissecting delicate nerves and frequent anatomical variations, including mere visual dissection without magnification in some autopsies, or investigation of only a few cadavers, which can bias the results.

## 3. Ways to Rebuild Breast Sensation

Various breast reconstruction methods exist following mastectomy, including implants, autologous tissue reconstruction, and hybrid techniques incorporating fat grafting. Autologous tissue reconstruction is considered the gold standard for breast reconstruction [11]. Among the various autologous techniques, the deep inferior epigastric perforator (DIEP) flap has emerged as the gold standard due to its superior tissue properties and preserved abdominal wall [12]. Although spontaneous recovery of sensation has been reported in the reconstructed breast, the results have been inconsistent and variable [13, 14]. The exact mechanisms of spontaneous flap sensory recovery have not been fully explored, and no studies have been conducted on the specific factors influencing spontaneous flap sensory recovery. Many studies suggest that the spontaneous recovery of sensation is limited, and sensory nerve grafting may offer a better and faster recovery of sensation in the breast [15]. Breast sensory nerve grafting should be pursued whenever possible.

### 3.1. Autologous Nerve Graft

When faced with a nerve defect, performing an end‐to‐end nerve anastomosis without tension is crucial. Tension during nerve sewing poses surgical challenges and risks fiber breakage. Tension‐induced nerve injuries suffer from inadequate blood supply, hypoxia, and increased scarring. Achieving tension‐free repair is not always possible due to factors like nerve stump retraction, scarring, or tissue damage. In such cases, autologous nerve grafts are vital and serve as the gold standard for bridging peripheral nerve defects [16].

Autologous nerve grafts are commonly utilized in reconstructive procedures to address peripheral nerve deficiencies. Grafts from the common peroneal nerve have shown effectiveness in facial nerve grafting for treating facial paralysis, as well as in nerve lengthening surgeries for brachial plexus injuries, with encouraging results [17, 18]. Corneal sensation is restored by employing functional lengthening of the suprasellar or supraorbital sensory nerve and redirecting its axons to the affected cornea [19]. Lateral antebrachial cutaneous nerve grafts are used to neurotize the cornea, among others [20].

Autologous nerve grafts are employed in breast reconstruction primarily using skin flaps with sensory nerves. Since 1887, when French surgeon Aristide first employed autologous tissue for breast reconstruction, autologous breast reconstruction has evolved to incorporate pedicled contralateral breast, free lipoma transplantation, pectoralis minor muscle flap, pedicled latissimus dorsi myocutaneous flap, free superior gluteal artery myocutaneous flap, free transverse rectus abdominis myocutaneous flap, and deep inferior epigastric artery perforator [21]. Autologous flap breast grafting has evolved significantly, initially prioritizing aesthetic outcomes over neurological reconstruction. With advancing surgical techniques and growing patient demands, there is a heightened focus on restoring breast sensation during reconstruction procedures. Numerous flaps with sensory nerves have been employed for breast reconstruction.

The use of each of these autologous nerve grafts has facilitated some degree of sensation improvement in the reconstructed breast, but optimal sensory recovery was not achieved. In Ducic’s study, separating the sensory and motor branches of the DIEP flap and anastomosing only the sensory branch to a recipient chest nerve resulted in superior sensory recovery in the reconstructed breast. The study also demonstrated that mixed nerve grafts impeded maximal sensory recovery from nerve grafts. Better outcomes may be obtained by performing only sensory branch nerve grafts rather than mixed grafts at the time of grafting [22]. However, it should be noted that this is a descriptive anatomical study with a relatively small sample size. In Juan’s randomized controlled clinical trial study, the selection of appropriately sized second to fourth ICNs, their dissociation, and anastomosis to the areolar tissue resulted in significant improvements in postoperative patient sensation and quality of life. This innovative approach, which utilized exclusively autologous nerves from the same surgical site, reduced the risk of immune rejection and donor area morbidity, marking a significant advancement in breast autologous nerve grafting techniques [23]. Chang utilized the LCB of the 4th ICN for breast nerve grafting in NSM, skin‐sparing mastectomy, delayed reconstruction with nipple preservation, and delayed reconstruction without nipple preservation, resulting in significant restoration of breast sensation. This retrospective case‐control study has expanded the possibility of performing autologous nerve grafting breast reconstruction across a broader range of patient populations [24].

Currently, the DIEP flap stands as the gold standard for autologous breast reconstruction due to its favorable tissue characteristics, low donor site complications, and preserved abdominal wall function. However, not all patients are suitable candidates for breast reconstruction utilizing DIEP flap. The optimal donor nerve and recipient nerve for breast reconstruction remain controversial. The selection of the appropriate flap and nerve should be individualized based on patient characteristics when performing breast nerve grafting.

### 3.2. Allogeneic Nerve Graft

Autologous nerve grafts, while advantageous, are subject to certain limitations, including donor site morbidity, potential size disparities, and the finite availability of donor tissue. Decellularized allogeneic nerve grafts have recently become widely used as an alternative to autologous nerve grafts with favorable results. In a retrospective, observational, multicenter registry study, Rinker investigated the use of processed nerve allografts (PNAs) for repairing nerve injuries greater than 25 mm in the hand. These results demonstrate that outcomes for PNA repairs of digital nerve injuries with gaps longer than 25 mm compare favorably with historical reports for nerve autograft repair, but without the donor site morbidity [25]. Allogeneic nerve grafts are now extensively employed in the repair of peripheral nerve defects. For example, facial nerve grafting using cell‐free allogeneic nerve grafts has shown rapid and complete recovery [26]. Good results have been achieved with upper extremity nerve repair using treated allogeneic nerve grafts [27]. Satisfactory outcomes have been achieved in the resection and reconstruction of symptomatic neuromas using treated allogeneic nerve grafts, etc., [28].

Allogeneic nerve grafts have also proven successful in breast nerve grafting. In a prospective cohort study, Momeni performed flap nerve grafting using treated human allogeneic nerve grafts and showed that this approach restored better protective breast sensation after ≥ 12 months of follow‐up compared with those without nerve grafting [29]. Ducic performed breast reconstruction utilizing DIEP flap, carefully separating the sensory and motor branches of the ICN in the flap and preserving the flap with only sensory branches [22]. They connected donor and recipient nerves using treated human allogeneic nerve grafts, employing different combinations. This approach optimizes the restoration of breast sensation. In immediate implant breast reconstruction, Peled meticulously dissected to identify and preserve the 4th and/or 5th ICNs at the thoracic cage whenever possible. Nerve grafting was then performed using a 1‐2 × 70 mm Avance allogeneic nerve graft. Connector‐assisted allogeneic nerve grafts to proximal nerve terminal wraps or 9‐0 external nerve sutures were performed using 8‐0. Once the implant was in place, a distal nerve wrap was performed on the previously identified subareolar nerve, and the allogeneic nerve graft was carefully placed directly over the acellular dermal matrix. This prospective case series showed that over 90% of patients reported good sensation in both the skin envelope of the breast and the NAC [30]. In a retrospective cohort study, Tevlin dissected the preserved lateral ICN to a certain length, wrapped the ICN to a nerve graft, and then wrapped the distal nerve graft to the nerve stump at the base of the NAC through a free flap tunnel [31]. This technique enhanced the recovery of sensation in the NAC. In a prospective case series, R. Djohan, for the first time, connected the anterior branch of the donor 4th lateral ICN to the target NAC with a treated nerve graft in implant‐based reconstruction [32]. The results showed a tendency to restore sensation in the NAC after implant reconstruction. This is the first study to report on early results obtained after performing sensate implant‐based breast reconstruction. More studies are required to determine the long‐term outcomes and impact on quality of life and to assess whether patient or breast characteristics impact the success of this procedure. Gfrerer used allogeneic nerve grafts to attach multiple donor nerves (LCBs of the 3rd to 5th ICNs) to the NAC in gender‐affirming mastectomy [4]. The study demonstrated restoration of sensation in most patients, including sexual sensation in some patients. This surgical technique report demonstrates the technical feasibility of using processed nerve allogeneic nerve grafts for multibranch nerve coaptation. Future studies with larger sample sizes and more rigorous designs, such as prospective cohort studies, are required to evaluate its efficacy in sensory recovery.

Allogeneic nerve grafts facilitate overcoming size mismatches more readily than autologous nerve grafts. The laborious process of dissecting the required anatomical length of both donor and recipient nerves, as well as the subsequent complications, is mitigated [33, 34]. A single allogeneic nerve graft can be divided into fascicles to connect multiple donor ICNs and cover the dermatosensory peripheral nerve elements of the dermis beneath the NAC. This approach maximizes the number of donor axons and the area of reinnervation. There is better recovery of breast sensation after surgery [31]. In a gender‐affirming mastectomy, a single ICN of adequate length was dissected and sutured directly to the NAC. The distal nerve graft end is split into fascicles, rather than being placed on the dermis as one unit. When grafts are placed as a single unit, all axons converge in one small area, which may result in focal hypersensitivity. Spreading out fascicles may increase the reinnervation zone and prevent hypersensitivity of the target skin. With a large number of fascicles entering the nerve graft, a sufficient number of axons should reach each fascicle and the final target [4, 35]. The literature on peripheral nerves indicates that donor axon counts are associated with the recovery of function and sensation after nerve grafting [36]. Allogeneic nerve grafts have streamlined breast nerve grafting, facilitating sensation reconstruction in the NAC. However, some of the disadvantages of allogeneic nerve grafts have also limited its widespread use. For example, a major issue regarding allogeneic nerve graft is host immune tolerance [37]. The maximum length of allogeneic nerve grafts that can be used for transplantation remains unknown. In a multicenter prospective cohort study, Brooks et al. reported that allogeneic nerve grafts bridge defects of 0.5–5.0 cm [38]. In a multicenter prospective registry study, Safa et al. concluded that the length of allogeneic nerve grafts bridging the neural gap varied between 1.5 and 7.0 cm [39].

Autologous nerve grafts are characterized by optimal biocompatibility, eliminating the need for immunosuppression, and yield reliable sensory outcomes. Their primary drawback, however, is donor site morbidity, which becomes particularly significant in patients with multiple nerve injuries or limited donor nerve availability. The principal advantage of allogeneic nerve grafts is characterized by the elimination of donor site morbidity, simplification of the surgical procedure (confined to a single surgical site), and the provision of a valuable solution when donor nerves are scarce. Their primary disadvantages, however, include high costs and the risks associated with the requisite short‐term immunosuppressive therapy. A comparison between autologous nerve grafts and allogeneic nerve grafts is presented in Table 1.

**Table 1:** Comparative analysis: autologous nerve grafts vs. allogeneic nerve grafts.

Aspect of comparison	Autologous nerve graft	Allogeneic nerve graft
Donor site morbidity [40]	Significant. Harvesting a donor nerve creates a second surgical site, resulting in potential sensory loss, neuroma formation, scar, and prolonged operative time.	None.

Immune risk [40]	None. As an autologous tissue, it is nonimmunogenic and offers optimal biocompatibility.	Requires management. The graft is processed to remove immunogenic cellular components (Schwann cells, axons), but the extracellular matrix may elicit a minor immune response. This typically necessitates a short‐term systemic immunosuppressive regimen, introducing associated risks and compliance considerations.

Cost [41]	Lower graft cost, but higher indirect costs. No expensive graft material is required. However, prolonged operating time for harvest and preparation increases room and anesthesia charges. Subsequent management of donor site complications may also add costs.	High material cost. The allogeneic nerve grafting is a significant expense. This may be partially offset by reduced overall operative time and the avoidance of costs associated with managing donor site morbidity.

Sensory outcome [25]	Gold standard, reliable. Extensive long‐term clinical evidence demonstrates consistent and meaningful recovery of sensory and motor function. It remains the benchmark against which other techniques are measured.	Comparable, but immunosuppression‐dependent. Numerous studies indicate recovery of meaningful sensory and motor function is equivalent to autologous nerve grafts for gaps typically up to 50–70 mm. Successful outcomes are contingent upon patient compliance with the immunosuppressive regimen during the critical period of regeneration.

### 3.3. Hybrid Nerve Conduit Graft

Nerve guidance conduits (NGCs) as an alternative to autologous nerve grafts have been widely used in recent years, especially in peripheral nerve defects. The NGCs, comprising biologic or abiotic material, serves as a prefabricated tube housing both distal and proximal nerve ends. Adherence of the outer nerve membrane to the tube’s wall facilitates axonal growth from the proximal to the distal end within the lumen. The material sources of NGCs mainly consist of natural macromolecules, such as silk fibroin, collagen, and chitosan, as well as synthetic polymeric materials such as polylactic acid, polycaprolactone, and polylactic‐co‐glycolic acid (PLGA) [42]. The food and drug administration (FDA) has approved three types of bioresorbable catheters made of caprolactone, collagen, or polyglycolic acid (PGA). Caprolactone catheters have been found to be comparable to autologous nerve grafts in terms of outcomes. Collagen catheters are the next best, and PGA catheters are functionally inferior [43]. Many nerve grafts have been developed in the form of NGCs [44], such as conductive NGCs based on morpho butterfly wings for peripheral nerve repair, functional polymer NGCs for peripheral nerve repair and regeneration, NGCs with hierarchical anisotropic structure for peripheral nerve regeneration, etc. [45–47].

Biologic, autogenous conduits—typically veins or, rarely, arteries—have demonstrated their utility in nerve gaps < 3 cm in length. In 1990, Chiu et al. reported a successful prospective series of autogenous vein nerve conduits compared with nerve autografts for digital nerve gaps ≤ 3 cm in length [48]. In a retrospective case series, Mackinnon bridged clinical nerve gaps of ≤ 3 cm with biodegradable PGA in 1990 [49]. There is a generally accepted upper limit of 3 cm on nerve conduit length. Most reported series of nerve conduits for reconstruction of digital nerve defects adhere to the 3 cm limit. Strauch et al. in a rabbit peroneal nerve study that compared results of axonal regeneration using vein conduits from 1 to 6 cm in length found excellent growth and function ≤ 3 cm, with deteriorating results at lengths > 3 cm [50]. However, as this is an animal model study, caution is warranted when extrapolating the findings to clinical applications. Inherent limitations to consider include species differences, the constraints of the disease model itself, divergent immune responses, and disparities in functional assessments. The clinical applicability of nerve conduits in terms of repair length is limited by several underlying biological factors, including the spatiotemporal distribution of neurotrophic factors, the migratory capacity of Schwann cells, and the intrinsic regenerative potential of axons.

In Spiegel’s prospective cohort study, DIEP flap nerve grafting using the third anterior ICN is an effective technique to provide a significant increase in sensory recovery for breast reconstruction patients, while adding minimal surgical time. The use of a nerve conduit produces increased sensory recovery when compared to direct coaptation [33]. In a retrospective cohort study, Djohan has achieved success in breast reconstruction using a novel autologous nerve graft technique for breast reconstruction using a processed allogeneic nerve graft combined with a NGC in an abdominal free flap [51]. However, the ≤ 3 cm gap restriction of NGCs limits their utility in breast reconstruction, particularly for NAC reinnervation over longer distances [43].

The role of the NGC is to provide temporary access to the regenerating nerve, and after the nerve fibers have passed through the conduit to the distal end, the NGC no longer has any effect, but may have a negative effect on the regenerating nerve fibers. This is directly related to the prognosis of nerve function recovery. It is best to use biodegradable materials to prepare the nerve conduit. Biodegradable artificial materials possess the advantage of biodegradability, thereby eliminating the need for a secondary surgical procedure for removal. These structures are easily shaped, elastic to reduce nerve compression, and simple to manufacture. Although biodegradable synthetic materials have a wide range of clinical applications, which have been approved by the FDA for clinical use, they lack cellular recognition signals and do not interact with cells in a biologically specific manner. The acidic byproducts of degradation lower the local pH around the graft, triggering sterile inflammation and negatively affecting cell and tissue growth. Additionally, their slow degradation rate increases the risk of post‐implantation fibrosis and may provoke immune reactions in surrounding tissues, limiting their clinical application [52].

## 4. Breast Sensory Evaluation After Breast Reconstruction

Objective sensory assessments of the breast encompass pressure, touch, pain, cold, heat, two‐point discrimination, and vibration. The commonly used methods for assessing breast sensation are listed in Table 2. Semmes–Weinstein monofilament examination and BREAST‐Q scale currently represent the most reproducible and commonly employed measurement methods in clinical research [53].

**Table 2:** Methods of assessing breast sensation.

Relatively objective sensory assessments	Subjective sensory assessments
Semmes–Weinstein monofilament examination [29]	BREAST‐Q scale [53]
von Frey test for quantifying skin pressure threshold [54]	The British Medical Research Council sensory function scale
Pressure‐specified sensory device [55]	Mackinnon–Dellon scale
Weber static two‐point discrimination test	Modified Mackinnon–Dellon scale
Dellon dynamic two‐point discrimination test	
Somatosensory‐evoked potentials (SEP) [55]	
The sharp and blunt discriminatory test	
Tactile and thermal quantitative sensory testing (QST) [55]	

Using a pressure‐specified sensor to assess sensory recovery offers several advantages over the Semmes–Weinstein monofilament test. This device is sensitive and specific, quantifies pressure thresholds for both static and dynamic touch, and exhibits reproducibility between patients and time points [56]. However, limited clinical availability restricts its utilization. Most tests can identify sensory nerve damage postoperatively, with tactile and thermal QST being the most reliable and sensitive for confirming sensory recovery [55]. SEP recording is useful for differentiating surgical techniques, while epidermal nerve fiber density and clinical examination perform poorly, except for the sharp–blunt discrimination [55]. Subjective sensory measures rely on patient self‐evaluation after breast surgery. The assessment of sensory recovery in patients after breast reconstruction is influenced by numerous factors, so a combination of multiple measurement instruments and both subjective and objective measurements should be used to assess sensory recovery whenever possible. The laborious dissection of donor and recipient nerves, along with associated complications, is mitigated.

## 5. Sequence of Sensory Recovery and Zonal Assessment in Breast Reconstruction

Numerous methods exist for zonally assessing the breast. Figure 1 summarizes the zonal approach to measuring breast sensation. In the study by Dossett, the breast compartmentalization measurements of Figure 1(a) were used [57]. Djohan is measured as shown in Figure 1(b) [51]. Momeni’s measurements are shown in Figure 1(c) [29]. The approach used in Khan’s study is shown in Figure 1(d) [58]. For the sensory measurement of breast reconstruction skin, theoretically the more areas measured the better, but the specific practical workload is too large to achieve, so it becomes important to select representative areas for testing. At present, there is no standardized measurement area, so we can refer to previous work to choose the way that satisfies our own study as much as possible.

Figure 1Breast sensation assessment modalities. (a) The breasts were divided into four quadrants (upper inner, upper outer, lower outer, and lower inner) and each of the five monofilaments was tested and recorded in the center of the quadrant, S being the skin around the breast. (b) Tested: (1) flap skin (skin paddle) and (2) mastectomy skin. The mastectomy skin was defined as 3 cm from the NAC. In cases of NAC absence, the most projected portion of the breast was used as a reference for the test. Both areas were divided into four quadrants. Thus, a total of nine points were tested in each of the areas, including four dynamic (quadrants) and five static tests (superior, medial, inferior, lateral, and flap center). (c) The breasts were divided into four quadrants by two lines; a vertical line was drawn from the mid clavicle to the nipple; a horizontal line was drawn perpendicular to the first line at the nipple level. The two lines intersected with a circle 3 and 1 cm from the edge of the areola, resulting in 8 points of intersection, plus the nipple, for a total of 9 points. (d) Schematic diagram of the six points examined in each breast during quantitative sensory testing. 1 is the upper outer quadrant, 2 is the upper inner quadrant, 3 is the lower inner quadrant, 4 is the lower outer quadrant, 5 is the point on the areola, and 6 is the point on the nipple.

**Figure:**
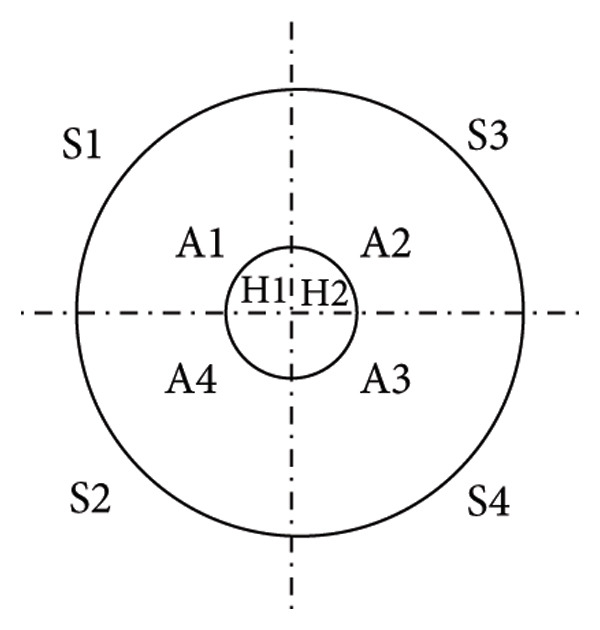
(a)

**Figure:**
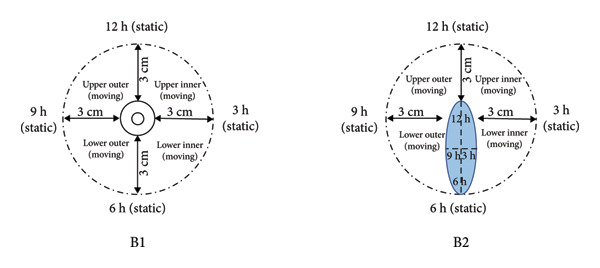
(b)

**Figure:**
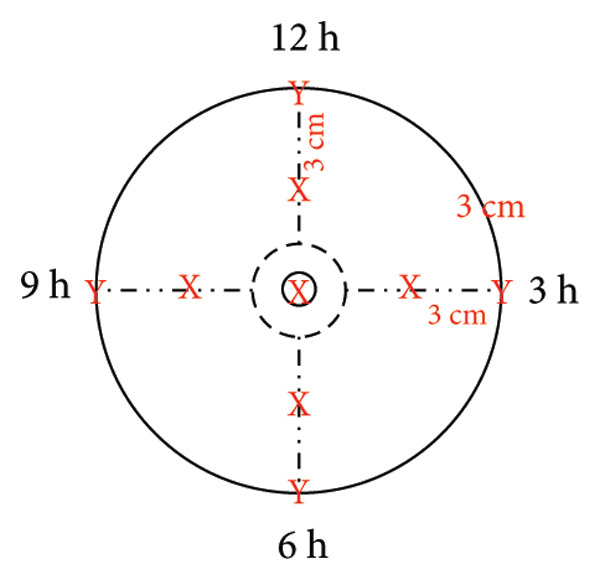
(c)

**Figure:**
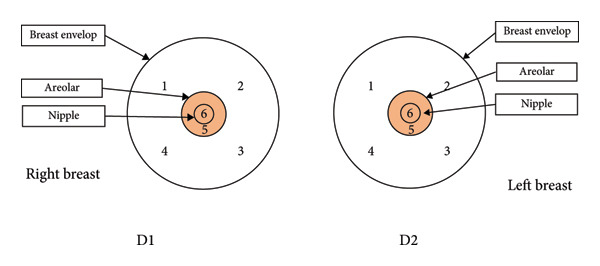
(d)

Sensory recovery varies in each quadrant, a prospective cohort study documenting slow sensory recovery in the outer upper quadrant [55]. Another prospective case series demonstrated superior sensation in the inner upper quadrant for mastectomy and NAC skin compared to other areas [59]. In a cross‐sectional study, Khan et al. demonstrated that sensory preservation was highest in the medial breast mound [58]. In a prospective cohort study of nerve injury during breast surgery, skin sensitivity was restored in the following sequence: pain, pressure, temperature, and two‐point discrimination [60]. Although nerve grafting can improve sensation after breast reconstruction, measurement of breast sensory recovery remains imprecise due to limitations of current measurement tools, etc. Further research is necessary to determine the sequence of breast sensory recovery after nerve grafting.

## 6. Factors Affecting Sensory Recovery

Factors influencing sensory recovery post‐nerve grafting remain nascent in their exploration. Early explorations into breast sensory recovery were rooted in the findings from hand nerve grafting, underscoring the significance of promptly reinstating nerve continuity and coherence. Creating a favorable microenvironment for nerve regeneration requires techniques such as suturing, nerve grafting, and administering neurotrophic agents. Disturbances in the microenvironment and the adhesion of adjacent tissues can affect nerve regeneration and functional recovery. Numerous factors come into play, including the location of nerve disruption, the timing of reconstruction, the extent of the nerve gap, as well as age and smoking habits, all of which impact the ultimate outcome [61]. Table 3 provides a summary of the factors influencing the effectiveness of nerve grafts. The most critical factors include surgical techniques, sources of nerve grafts, time, and the extent of nerve damage. Secondary factors encompass biological factors, age, post‐transplant rehabilitation and neural stimulation, postoperative certificates of concurrent conditions health condition, immune response, and innovations in nerve regeneration technology.

**Table 3:** Factors affecting the effectiveness of nerve grafts.

Type	Factor	Consequence
Sources of nerve grafts	Autologous nerve grafts	Superior biocompatibility, reduced rejection, and improved recovery outcomes [62].
	Allogeneic nerve grafts	Increased risk of rejection [63].
	NGC	Biodegradable scaffolds facilitate nerve fiber growth and enhance regeneration [64].

Surgical techniques	Precision of nerve repair	Precise microsurgical suturing and accurate gap alignment are crucial for regeneration, especially in minimally invasive surgery [65].

Biological factors	Nerve growth factor	Application facilitates growth and repair, enhancing graft efficacy [66].
	Regenerative environment	Blood supply, inflammation, and extracellular matrix state critically influence recovery quality [67].

Post‐transplant rehabilitation	Physical stimulation and therapeutic training	Post‐surgical rehabilitation preserves nerve fibers and promotes neuronal connections [68].
	Strategies for pain management	Effective pain management is crucial for optimizing recovery [69].

Interindividual differences	Age	Regeneration ability declines with age [70].
	Health condition	Overall health impacts success [51].
	Extent of nerve damage	Regeneration potential is inversely correlated with injury severity [71].

Postoperative considerations	Nerve scarring	Scarring hinders fiber regeneration and impedes recovery [72].
	Nerve overgrowth	Overgrowth may cause neuropathic pain or allergic reactions [73].

Time	Time delay for transplantation	Timely grafting is critical for maximizing recovery [74].

Immune response	Immune response	Immune rejection hinders regeneration. Immunosuppressants can alleviate this issue [75].

Innovations in technology	Application of biomaterials	Bioscaffolds and nanotechnology enhance growth guidance and improve graft success rates [76].

Some studies suggest the superiority of autologous nerve grafts over allogeneic variants. This observed bias may stem from technical, individual, and methodological differences. Contributing factors include surgical technique and timing, patient‐specific variables, graft biology, as well as common study limitations such as retrospective designs, small sample sizes, and the lack of standardized sensory assessment. Given the complexity involved in optimizing all variables affecting nerve regeneration, achieving full functional recovery after nerve grafting reconstruction remains an exception rather than the norm. It is crucial to identify and address factors that may negatively influence nerve regeneration as much as possible prior to surgery, in order to guide patient selection and manage the expectations of both patients and surgeons.

## 7. Outlook

The reconstruction of breast sensation after mastectomy for breast cancer is of paramount importance. Nerve grafting techniques in breast reconstruction are still under development and refinement. While it has yielded positive results in many cases, challenges persist regarding the technical aspects, postoperative outcomes, patient‐specific factors, lack of standardized sensory tests, and small clinical cohorts. In the future, as medical technology and science continue to advance, it is anticipated that more sophisticated and effective nerve grafting techniques will be employed in clinical practice. The reconstruction of a beautiful and sensitive breast is no longer a distant dream but a tangible possibility.

## Ethics Statement

The extrapolation of data from animal models to clinical applications is associated with several inherent limitations that must be considered, including species differences, the limitations of disease models, disparities in immune responses, and gaps in functional assessment.

The use of allogeneic nerve grafts in human clinical research raises several ethical considerations. A primary concern is that the tissue donor source must be based on explicit, voluntary informed consent and adhere to national and international ethical guidelines for tissue donation. Alongside this, issues of safety and social justice in human subjects research, the balancing of risks versus benefits, and the necessity for long‐term follow‐up and data transparency must also be addressed.

## Disclosure

The manuscript is approved by all authors for publication.

## Conflicts of Interest

The authors declare no conflicts of interest.

## Author Contributions

Yan Shen: writing–original draft. Juan Zhang and Yihua Liu: writing–review and editing. Haifeng Cai: writing–review and editing and supervision.

## Funding

No funding was received for this manuscript.

## Data Availability

We conducted a comprehensive narrative review. An electronic search of the literature was performed using the PubMed, Embase, and Scopus databases. The search strategy incorporated a combination of keywords and Medical Subject Headings (MeSH) terms, including “breast reconstruction,” “nerve grafting,” “sensory recovery,” “neurotization,” and related terms. Literature selection was based on the authors’ expert judgment, prioritizing clinical studies, technical reports, and review articles relevant to the application and outcomes of nerve grafting in breast reconstruction. Data supporting this review’s conclusions are available from the corresponding author upon request.
